# The Potential Impact of Improving Appropriate Treatment for Fever on Malaria and Non-Malarial Febrile Illness Management in Under-5s: A Decision-Tree Modelling Approach

**DOI:** 10.1371/journal.pone.0069654

**Published:** 2013-07-29

**Authors:** V. Bhargavi Rao, David Schellenberg, Azra C. Ghani

**Affiliations:** 1 Medical Research Council Centre for Outbreak Analysis & Modelling, Department of Infectious Disease Epidemiology, Imperial College London, London, United Kingdom; 2 Disease Control and Vector Biology Unit, Department of Infectious and Tropical Diseases, London School of Hygiene and Tropical Medicine, London, United Kingdom; University of Barcelona, Spain

## Abstract

**Background:**

As international funding for malaria programmes plateaus, limited resources must be rationally managed for malaria and non-malarial febrile illnesses (NMFI). Given widespread unnecessary treatment of NMFI with first-line antimalarial Artemisinin Combination Therapies (ACTs), our aim was to estimate the effect of health-systems factors on rates of appropriate treatment for fever and on use of ACTs.

**Methods:**

A decision-tree tool was developed to investigate the impact of improving aspects of the fever care-pathway and also evaluate the impact in Tanzania of the revised WHO malaria guidelines advocating diagnostic-led management

**Results:**

Model outputs using baseline parameters suggest 49% malaria cases attending a clinic would receive ACTs (95% Uncertainty Interval:40.6–59.2%) but that 44% (95% UI:35–54.8%) NMFI cases would also receive ACTs. Provision of 100% ACT stock predicted a 28.9% increase in malaria cases treated with ACT, but also an increase in overtreatment of NMFI, with 70% NMFI cases (95% UI:56.4–79.2%) projected to receive ACTs, and thus an overall 13% reduction (95% UI:5–21.6%) in correct management of febrile cases. Modelling increased availability or use of diagnostics had little effect on malaria management outputs, but may significantly reduce NMFI overtreatment. The model predicts the early rollout of revised WHO guidelines in Tanzania may have led to a 35% decrease (95% UI:31.2–39.8%) in NMFI overtreatment, but also a 19.5% reduction (95% UI:11–27.2%), in malaria cases receiving ACTs, due to a potential fourfold decrease in cases that were untested or tested false-negative (42.5% vs.8.9%) and so untreated.

**Discussion:**

Modelling multi-pronged intervention strategies proved most effective to improve malaria treatment without increasing NMFI overtreatment. As malaria transmission declines, health system interventions must be guided by whether the management priority is an increase in malaria cases receiving ACTs (reducing the treatment gap), reducing ACT waste through unnecessary treatment of NMFI or expanding appropriate treatment of all febrile illness.

## Introduction

Malaria remains a major public health problem, with an estimated 216 million cases and 655,000 deaths in 2010 [1]. In endemic areas, a significant proportion of clinic visits and hospital admissions relate to malaria [1], [2], with severe disease and mortality often ensuing from delayed or inadequate treatment [3]–[7]. Recent scaling-up of malaria control programmes has led to reductions in reported malaria cases, albeit slower than internationally agreed targets for 2010 [1]. However international funding for control programmes is expected to plateau, and fall to levels lower than required to meet such targets [1], [8]. As such, it is essential that limited resources, including first-line treatments such as Artemisinin Combination Therapies (ACTs), are rationally managed.

Until recently presumptive treatment and syndromic management of all fevers as malaria was advocated in WHO guidelines and national policies, especially for children under 5 years (U5s). This has resulted in overtreatment (unnecessary prescription of antimalarials) with 47%–95% of patients with non-malarial febrile illness (NMFI) estimated to receive antimalarials [9]–[17]. Overtreatment is often with non-recommended antimalarials [11], [18], but may also involve first-line ACTs [9], [11], [12], [15], [17]. The latest 2010 WHO guidelines revised protocols for the treatment of malaria and state that whenever possible “*prompt parasitological confirmation by microscopy or alternatively by rapid diagnostic test (RDT) is recommended in all patients suspected of malaria before treatment is started. Treatment solely on the basis of clinical suspicion should only be considered when a parasitological diagnosis is not accessible*” [19]. This policy change was adopted to reduce routine overtreatment of malaria and the consequent risk of drug resistance, to expand disease surveillance and to improve quality of care for both malaria and NMFI, though its likely impact remains a subject of debate [20]–[24]. The WHO estimate expenditure on treatment may decrease as a result of testing before treatment and reduced prices for RDTs and ACTs [1], although any reduction in first-line drug costs could be complicated by a rise in anti-malarial resistance requiring alternative antimalarials [25]–[27]. However non-compliance with test results by healthcare workers (HCWs), i.e. treating with antimalarials despite a negative test for malaria, is common [28]–[30] and can be detrimental to those patients who are not parasitaemic. For example, a Tanzanian study found the case fatality rate in test-negative patients treated with antimalarials to be significantly higher (12.1%) than for test-positive patients (6.9%), and over 60% of NMFI were not treated with antibiotics [10].

The sustainability and efficiency of malaria control is limited by the capacity of impoverished health systems to deliver interventions at the required levels of coverage and quality, ensuring those who need treatment receive it, and that those who do not are not needlessly treated. There have been relatively few modelling approaches to address the delivery of treatment for case management. The “systems effectiveness framework” [31] illustrates how interacting health-systems barriers may sequentially reduce the in-field effectiveness of treatment interventions [32], [33]. This has proved valuable as a means of analysing the steps to optimal case management. However outcomes such as the proportion of malaria cases that receive first-line treatment through all pathways (i.e. not solely via diagnostic-led management) and the levels of unnecessary treatment of NMFI with antimalarials are not addressed by this approach. Such outcomes are important given the limited budgets for the purchase and distribution of antimalarial treatment courses. Data from the INESS trial in Ghana, using this framework, estimates that just 13.5% of simple malaria fevers are treated effectively, with the greatest loss due to failure to access care within 24–48 hours [34]. Patient adherence was included in this analysis and constituted the second largest bottleneck [34]. However this differs from WHO estimates of cases of malaria treated with ACTs and other published studies [35], [36] in some part because it does not include alternative non-recommended pathways to receiving treatment.

Here we extend the systems effectiveness framework into a decision-tree tool to estimate the effect of systems factors on rates of appropriate treatment for fever cases and appropriate use of ACTs. Decision-tree approaches have previously been used to consider the role of diagnostics in reducing the burden of childhood malaria in Africa [37], [38]. Here we include considerations of treatment seeking, diagnostic availability, use and quality, as well as ACT stock in order to compare interventions to improve case management in a context specific manner. We also use this tool to undertake an early evaluation of the impact of the revised WHO guidelines on treatment outcomes for malarial and non-malarial fever.

## Methods

### Systems Effectiveness and Decision-Tree Model

**Figure 1:**
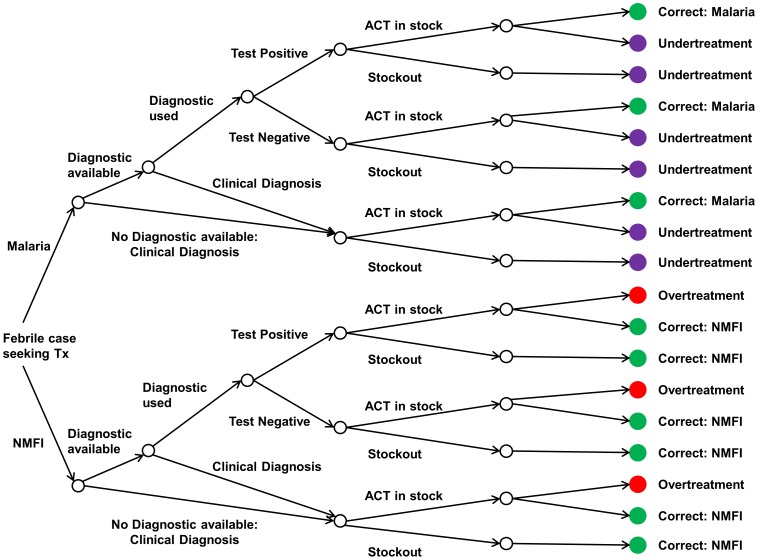
Decision tree modelling approach to malaria case management in the public sector. At the left-hand side the entry point is a febrile case seeking treatment. We next stratify on their true (unobserved) cause of fever as either malaria or non-malarial febrile illness (NMFI). The case management process then involves five steps – the availability of an RDT, whether the RDT is used, the outcome of the RDT given the true underlying cause of fever (based on the sensitivity and specificity of the diagnostic), whether an ACT is stock, and whether an ACT is prescribed given the RDT result or clinical diagnosis. This leads to four outcomes: correct treatment for malaria or for NMFI (shown as a green circle), under-treatment of malaria (shown as a purple circle), or overtreatment of an NMFI for malaria (shown as a red circle). In a perfect case management system there would be no under- or over-treatment.

We considered two approaches to evaluate the impact of improvements in case management on the appropriate treatment of fevers in U5 children in malaria endemic settings. The first follows the published stepwise systems effectiveness approach to case management [31], [32], [39], whilst the second is a decision-tree approach to malaria treatment in the public sector (Figure 1) extending previous similar decision-tree models for diagnostics [37], [38]. The entry point to both scenarios is a febrile case seeking treatment. Treatment following clinical (i.e. non-diagnostic guided) diagnosis is included in the decision tree model, but not in the published systems effectiveness framework.

The outcome of the systems effectiveness approach is the proportion of malaria cases that receive correct diagnostic-led treatment with ACTs. In contrast, the decision-tree approach allows a wider spectrum of outcomes to be evaluated: i) correct treatment of malaria with ACTs (diagnostic-led or clinically diagnosed), ii) the under-treatment of malaria cases (i.e. those not given ACTs), iii) overtreatment of NMFI with ACTs, and iv) the overall number of febrile patients treated appropriately (i.e. both malaria cases given ACTs and NMFI not treated with ACTs).

Staff availability and training in malaria management were not included at this stage as, despite having potential impact, their effects can be difficult to quantify [40]. Stockouts of treatment for NMFI were not considered given the diversity of possible bacterial and non-bacterial causes, uncertainty regarding the need for antibiotics, and the high likelihood of basic antibiotics being available. In addition since the focus here is the impact of the health system, patient adherence to ACTs prescribed and drug failure were not included in either model.

### Model Parameters

Model parameters for the moderate-high transmission setting analysis were derived from a previously published systematic literature review [40]. The parameters were restricted to data presented in studies published between January 2004 (following adoption of ACT as first-line treatment in most countries) and November 2012. The model parameters are shown in Table 1. For each health-systems parameter we extracted any relevant data from the papers restricting our analysis to medium-high transmission settings (as reported in the papers included), stratified by whether the study was conducted before or after the introduction of the WHO guidelines regarding universal rational (diagnostic-led) treatment in 2010 [19]. Parameters for diagnostic performance were derived from published values for the sensitivity and specificity of RDTs. We did not limit this to a specific type of RDT. We did not differentiate between the various types of RDTs or microscopy for parameters of diagnostic availability and use. Case management values for low prevalence scenarios were limited. We included studies published in regions outside Africa (including Afghanistan) and used the results to inform estimates of parameters for a density plot comparing medium-high to low prevalence settings.

**Table 1:** Parameter estimates for each process in the cascade and decision-tree models.

	Pre universal rational treatment guidelines	Post universal rational treatment guidelines	Pre universal rational treatment guidelines	Post universal rational treatment guidelines	References
	All Studies		Tanzania		
**Probability of seeking treatment at public sector clinic**	0.28 (0.26–0.39)	0.29 (0.26–0.40)	0.28 (0.26–0.39)	0.29 (0.26–0.40)	[35], [44]–[49]
**Probability fever is due to malaria**	0.22 (0.13–0.33)	0.22 (0.13–0.33)	0.18	0.1	[1], [15], [50]–[55]
**Probability that a diagnostic is available**	0.54 (0.36–0.97)	0.58 (0.50–0.83)	0.35 (0.34–0.36)	0.61 (0.55–0.68)	[9], [11], [12], [14], [17], [18], [35], [43], [44], [55]–[60]
**Probability that a diagnostic is used**	0.39 (0.29–0.58)	0.46 (0.34–0.46)	0.69 (0.47–0.71)	0.71 (0.52–0.83)	[11], [12], [14]–[17], [35], [36], [44], [55]–[59], [61]–[63]
**Diagnostic sensitivity**	0.90 (0.78–0.92)	0.86 (0.72– 0.92)	0.82 (0.63–0.92)	0.82 (0.62–0.86)	[59], [64]–[68]
**Diagnostic specificity**	0.86 (0.8–0.92)	0.91 (0.82–0.98)	0.89 (0.83–0.95)	0.98 (0.91–0.98)	[55], [59], [64]–[68]
**Probability that all doses of ACT are available**	0.65 (0.54–0.73)	0.64 (0.62–0.68)	0.59 (0.51–0.67)	0.85 (0.81–0.90)	[11], [12], [17], [18], [35], [43], [57]–[61], [69]–[75]
**Probability that ACT is received if test positive**	0.99 (0.91–1.0)	0.98 (0.76–0.99)	1.00 (0.99–1.00)	1.00 (0.87–1.00)	[11], [12], [16], [17], [35], [36], [56]–[59], [61]–[63], [65], [76]–[78]
**Probability that ACT is received if test negative**	0.51 (0.39–0.71)	0.25 (0.11–0.53)	0.77 (0.53–0.81)	0.12 (0.08–0.20)	[11]–[17], [35], [36], [55]–[59], [61]–[63], [65], [76], [77]
**Probability that ACT received if untested**	0.67 (0.65–0.84)	0.49 (0.23–0.71)	0.89 (0.79–0.95)	0.15 (0.08–0.21)	[11], [15]–[17], [35], [55]–[59], [61], [63], [74], [76], [77]

The values are stratified by whether the data were collected before or after the introduction of WHO diagnostic policy recommending universal diagnostic-led treatment for malaria. Values specific to a Tanzanian case study are also shown. The median and interquartile range from the published studies is presented. For the probability of seeking treatment at the public sector clinic, diagnostic sensitivity and diagnostic specificity, separate values for Tanzania were not available and so the general parameters were used. The probability of fever being due to malaria was assumed the same in the aggregated analysis but set to reflect the reduction in malaria incidence seen in Tanzania. In the Tanzanian case study, the probability of at least one dose of ACT being in stock was used rather than the probability of all doses of ACT being in stock due to limited data on the latter.

For the baseline scenario we calculated the median of the extracted estimates for each parameter and the 25^th^ and 75^th^ percentiles for the parameter range. These ranges were chosen so as not to skew the results by sampling outliers. To generate uncertainty intervals we generated 1000 random parameter samples, drawing each parameter independently from a Uniform distribution between the 25^th^ and 75^th^ percentiles.

### Case Management Scenarios

Parameters based on the literature prior to the publication of the new WHO guidelines were used as a baseline scenario representing current practice, since the rollout of guidance is in its early stage. We then investigated how improving case management at different points along the patient care-pathway impacted on the four outcomes in the decision-tree model. Table 2 summarises the set of scenarios considered.

**Table 2:** Scenarios for improved malaria case management.

Scenario	Modified Parameters
**Baseline**	
**100% diagnostic availability**	Probability that a diagnostic is available = 1
**100% diagnostic use**	Probability that a diagnostic is used = 1
**100% ACT stock**	Probability that all doses of ACT are available = 1
**100% compliance with test results (i.e. treatment of test-positive only)**	Probability that ACT is received if test positive = 1
	Probability that ACT is received if test negative = 0
**Perfect diagnostic**	Diagnostic sensitivity = 1
	Diagnostic specificity = 1
**100% diagnostic availability & use**	Probability that a diagnostic is available = 1
	Probability that a diagnostic is used = 1
**100% diagnostic availability & ACT stock**	Probability that a diagnostic is available = 1
	Probability that all doses of ACT are available = 1
**100% diagnostic use and compliance with results**	Probability that a diagnostic is used = 1
	Probability that ACT is received if test positive = 1
	Probability that ACT is received if test negative = 0
**100% diagnostic availability, use & compliance**	Probability that a diagnostic is available = 1
	Probability that a diagnostic is used = 1
	Probability that ACT is received if test positive = 1
	Probability that ACT is received if test negative = 0
**100% diagnostic availability, use & compliance & ACT stock**	Probability that a diagnostic is available = 1
	Probability that a diagnostic is used = 1
	Probability that all doses of ACT are available = 1
	Probability that ACT is received if test positive = 1
	Probability that ACT is received if test negative = 0
**100% perfect diagnostic availability, use & compliance & ACT stock**	Probability that a diagnostic is available = 1
	Probability that a diagnostic is used = 1
	Diagnostic sensitivity and specificity = 1
	Probability that all doses of ACT are available = 1
	Probability that ACT is received if test positive = 1
	Probability that ACT is received if test negative = 0

Table 2 describes the scenarios used in the model to investigate the impact of improving case management at different points along the patient care-pathway. Scenarios of individual interventions e.g. 100% ACT stock were first considered and then combinations of interventions were studied. The health system parameters that are perfected in each scenario are defined here. The results from the decision tree model for each of these scenarios are shown in Figures 3, 4, 5.

We also performed the same scenarios using only data from Tanzania as a case-study, in order to compare published data from the early stages of the rollout of the new WHO guidance with modelled outcomes. Due to a paucity of published information, the values from all sub-Saharan Africa studies were used for the probability of seeking treatment at a public sector clinic. In addition, in the Tanzanian case-study, the probability of at least one dose of ACT being in stock was used rather than the probability of all doses of ACT being in stock due to limited data on the latter. Table 1 summarises the model parameters for the Tanzanian case-study.

## Results

**Figure 2:**
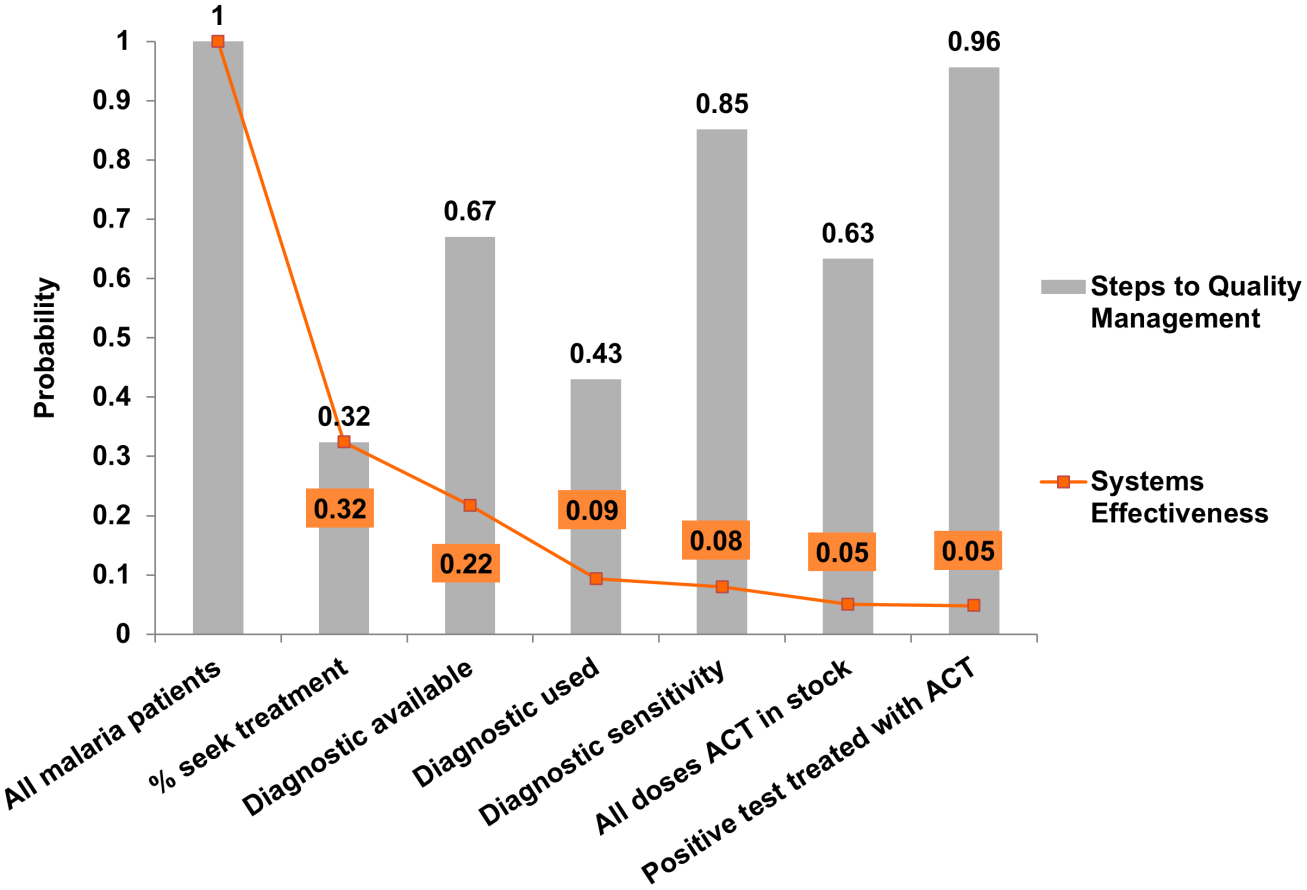
Estimated proportion of malaria cases at each case management point in the systems effectiveness pathway. The grey bars show the probabilities for each step for malaria case management whilst the orange line and values show the cumulative probability along this pathway. Data here is taken from studies published across sub-Saharan Africa prior to the rollout of the WHO guidelines on universal rational management.

Figure 2 shows the outcomes from the systems effectiveness model. Using the baseline parameters obtained from studies undertaken prior to the 2010 WHO guidelines on rational case management, we estimate that 4.7% (95% uncertainty interval [UI]: 2.1–8.8%) of all malaria cases, and 14.7% (95% UI: 6.9–25.6%) of those malaria cases that attend the health facility will be treated correctly. In contrast, using the decision tree model to account for the correct outcome being possible despite imperfect case management (e.g. a case may receive an ACT despite not being tested) we estimate that 54% (95% UI: 48.9–59.3%) of all febrile attendees in the public sector will be correctly managed, and that 49% of malaria cases attending a public facility would receive first line ACTs (95% UI: 40.6–59.2%). This is similar to the WHO estimate of malaria cases being treated with ACTs at health facilities [1] and hence appears to represent a rational model for case management evaluation. We also estimate that 44% (95% UI: 35–54.8%) of NMFI cases attending the clinic would unnecessarily receive an ACT.

We next used the decision-tree model to estimate the effect of improving the various case management steps alone and in combination. Increased treatment-seeking was the single most effective step in increasing the proportion of all febrile cases that would be correctly managed and all malaria cases receiving an ACT. Modelling 100% attendance at the facility resulted in 49.6% all malaria cases (95% UI: 40.9–58.63%) receiving an ACT compared with 16.2% (95% UI: 11.8–20.8%) at baseline. However this would have little anticipated effect in improving case management of those patients attending the clinic. Perfecting a single step in the care pathway almost always resulted in an overall predicted increase in the proportion of fever cases attending clinic that are correctly treated. The one exception was a scenario of improving ACT stock alone (100% availability), under which our model predicted a 13% point reduction (95% UI: 5–21.6%) in correct management of all febrile cases.

**Figure 3:**
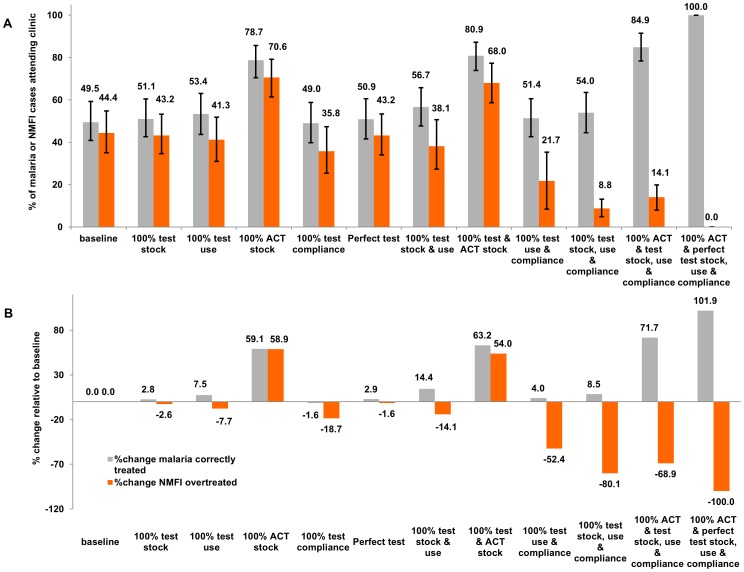
Results from the decision tree model for cases attending the health facility. A) % of malaria cases correctly treated with an ACT (grey bars) and % of non-malarial febrile illness (NMFI) overtreated with an ACT (orange bars) in a variety of scenarios as defined in Table 2 B) % change from baseline of malaria cases correctly treated with an ACT (grey bars) and % of non-malarial febrile illness (NMFI) overtreated with an ACT (orange bars) in each of the scenarios depicted in Figure 3A and defined in Table 2.

The breakdown of correct fever management into the proportion of malaria cases receiving an ACT and the risk of NMFI being over-treated with an ACT is shown in Figure 3, depicting the absolute percentage point and relative percentage change in these two outcomes predicted under a range of scenarios for improving case management steps. Provision of 100% stock of ACTs predicted a 28.9% point (95% UI: 20.5–36.1%) increase in the proportion of malaria cases given an ACT, which corresponds to a 59% increase relative to baseline. However, this was also accompanied by a 26% point (95% UI: 17.0–34.7%) anticipated increase in the overtreatment of NMFI, potentially resulting in 70% NMFI cases (95% UI: 56.4–79.2%) receiving an ACT. Thus the modelled decrease in correct management of all febrile cases in a scenario of 100% ACT stock is due to a larger proportion of NMFI cases predicted to receive ACTs since there is no limitation by drug stock.

Single interventions aimed at increasing availability or use of diagnostic tools were forecast to have little effect on improving the management of malaria cases or reducing NMFI overtreatment. Modelling perfect compliance with diagnostic results without any increase in diagnostic stock or use (i.e. positive tests treated with ACTs and negative tests not treated with ACTs) led to very little projected change in the proportion of malaria cases receiving an ACT but anticipated an 8.9% point reduction (95% UI: 2.6–19%) in NMFI overtreatment with ACTs (18% relative reduction). Improved diagnostic quality, (100% sensitivity and specificity) also led to small predicted improvements in malaria treatment and a decrease in NMFI overtreatment even when all other conditions were maintained at baseline. Combinations of improvements to diagnostics deployment however, may show an effect on NMFI management, for example, increasing the availability and use of diagnostics is predicted to reduce overtreatment of NMFI with ACTs to 38% (95% UI: 27.3–50.7%), constituting a 14% point reduction from baseline. This scenario also projected improved overall management of malaria cases, with 57% (95% UI: 47.7–65.8%) of malaria cases receiving ACTs, i.e. a 7% point increase (95% UI: −1.4–16.8%).

**Figure 4:**
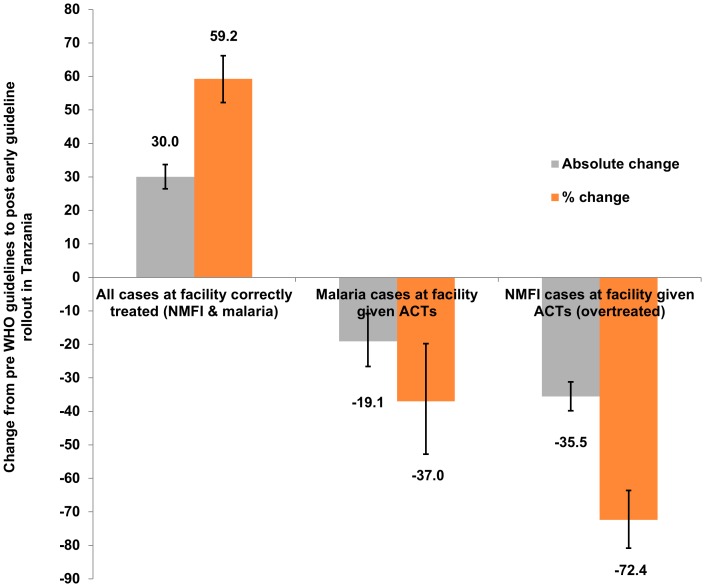
Change in case management outcomes after early rollout of WHO 2010 guidelines in Tanzania. The absolute percentage point change (grey bar) and percentage change relative to the baseline scenario (orange bar) following the introduction of the WHO 2010 case management guidelines advocating diagnostic-led treatment for all ages in Tanzania in 1) estimated proportion of attending cases correctly treated (both malaria and NMFI); 2) proportion of malaria cases correctly treated and 3) proportion of NMFI cases given an ACT. Data used were collected during the early period of the rollout of the new guidance and thus may not reflect more recent improvements in case management.

Using Tanzania as a case-study, we compared predicted case management outcomes using published before and after the 2010 WHO guidelines. The Tanzanian Malaria Indicator Study reported that malaria prevalence amongst U5s had dropped from 18% in 2007 to 10% in 2011 [41]. The data collected from studies published in the year following the guidelines rollout is summarised in Table 1, and indicates stock levels of any dose of ACTs had increased (from 59% to 85%) as well as availability of any diagnostic tools (from 35% to 61%). At this stage, levels of diagnostic usage were not seen to have substantially increased (69% compared to 71%), although compliance to test results had improved (the probability of receiving an ACT with a negative test result reduced from 67% to 14%) and treatment of untested cases had also decreased (86% untested febrile cases to 15%). Using these parameters in the decision-tree, Figure 4 depicts the predicted percentage change in the overall proportion of cases (both malaria and NMFI) correctly treated, the proportion of malaria cases correctly treated and the proportion of NMFI overtreated. The model estimates a 30% point increase (95% UI: 26.5–33.6%) in the proportion of all attending cases correctly treated would have occurred in the early stages of the implementation of the guidelines, i.e. a 52% relative increase compared with the pre-WHO guidance baseline. Contributing to this overall predicted improvement is a 35% point reduction (95% UI: 31.2–39.8%) in the proportion of NMFI treated inappropriately with ACTs, resulting in potentially only 13% of NMFI patients being overtreated following the guidance rollout. However we also predict a 19.5% point reduction (95% UI: 11 to 27.2%) may have ensued following rollout of the new WHO guidelines in the proportion of malaria cases receiving an ACT if they attend a clinic, i.e. 37.5% of attending malaria cases are given ACTs. Overall, on the basis of published health facility data from Tanzania, the percentage of all malaria cases in the community treated with ACTs is modelled to have reduced from 16.8% to 10.6%. Thus despite improved access to diagnostics, improved ACT stock and compliance to test results (but no increase in the overall proportion tested), the model outputs suggest a reduction in the proportion of malaria cases given ACT as treatment could have occurred. This does not mean that these malaria cases were not treated at all since we have not included other antimalarials aside from ACTs in our analysis. Exploration of the different pathways by which a malaria case may receive ACTs reveals a greater than twofold increase in the modelled probability of a malaria case being tested and receiving treatment on the basis of a positive test result (9.4% vs. 24%). However there is greater than fourfold reduction in the predicted probability of malaria cases receiving ACTs through other pathways (42.5% vs. 8.9%), i.e. in those untested or in those who falsely test negative and are hence untreated. Similar outputs are seen when comparing the combined dataset from all countries.

**Figure 5:**
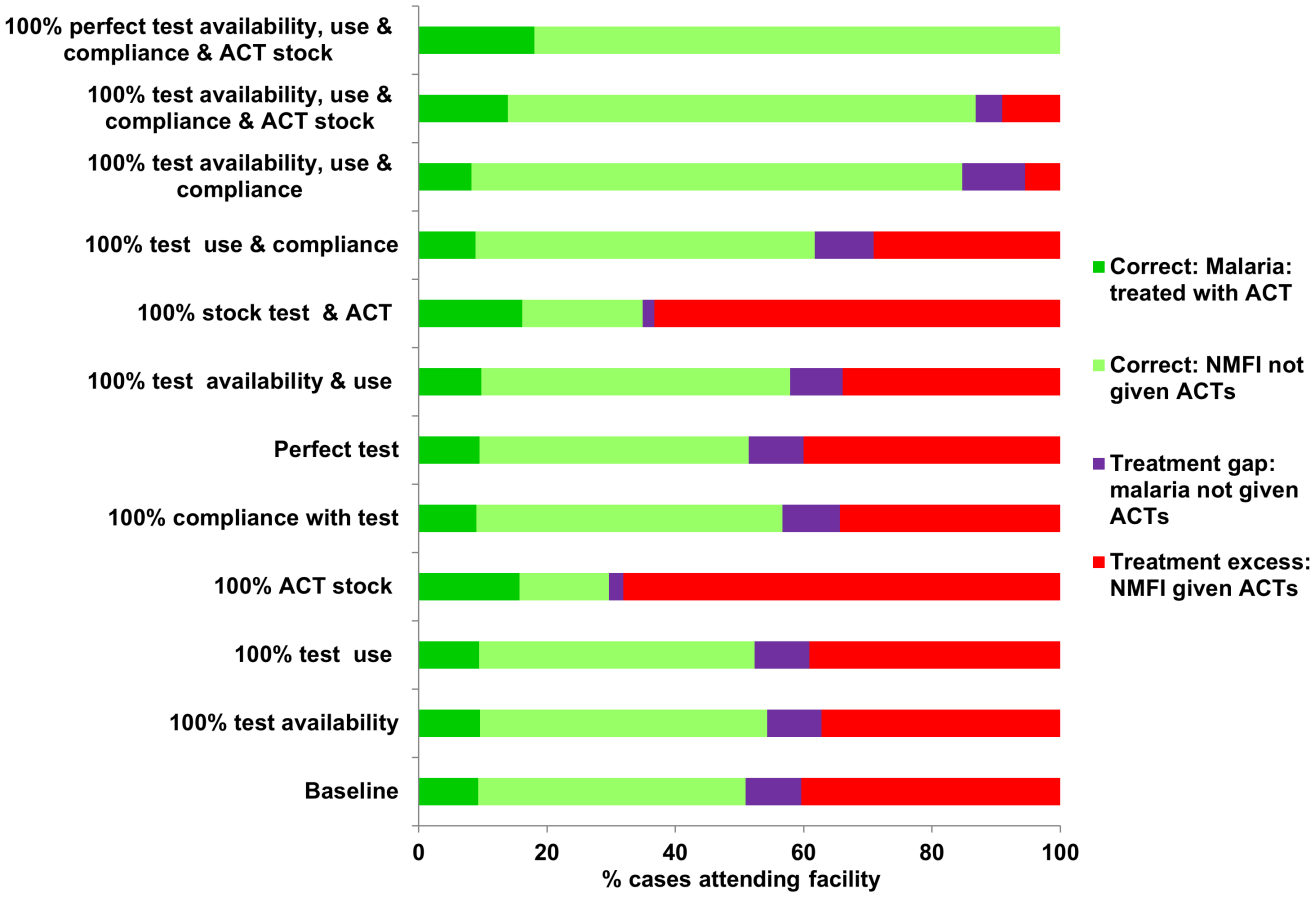
Modelled impact on treatment gap and treatment excess in Tanzania. Figure depicting the % treatment gap and % overtreatment (treatment excess) of all febrile patients attending health facilities using Tanzania as a case study, in the scenarios defined in Table 2. Desirable outcomes, namely malaria cases receiving ACTs and NMFI cases not being treated with ACTs are depicted in green. The % treatment gap, i.e. cases that need ACTs but that do not receive ACTs are depicted in purple. The % treatment excess i.e. cases that do not need antimalarials but are given ACTs unnecessarily are depicted in red.

Figure 5, using Tanzania data as a baseline case-study, shows the predicted gap between cases needing treatment and cases receiving treatment for the idealised scenarios investigated. At baseline we predict that there is a high degree of overtreatment, with less than a quarter of patients modelled to receive ACTs actually needing antimalarials, whilst there is a substantial treatment gap (i.e. malaria cases not given ACTs) with only half of patients needing antimalarial treatment forecast to actually receive ACTs. Overtreatment can be reduced by improving compliance with diagnostic results, as well as diagnostic availability; although a treatment gap may remain. In contrast high levels of ACT stock alongside high availability, use and compliance to diagnostic tests are predicted to reduce the treatment gap, i.e. increase the likelihood that those in need of treatment receive ACTs. However, this may also increase over treatment (i.e. NMFI given ACTs unnecessarily). If additionally the performance of the diagnostic test is improved (here we assume 100% specificity and sensitivity), the model output predicts no further treatment gap or treatment excess. Figure 5 illustrates the potential policy trade-off between increasing diagnostic use and compliance versus increasing ACT stock with respect to reducing the treatment gap and limiting treatment excess at medium-high transmission settings.

## Discussion

Our simple decision-tree model can provide insight into aspects of delivering care most likely to impact on care quality and programme efficiency, and can quantify the intuitive qualitative effects of refining different steps of the care pathway in order to help to inform decisions and guide investments in improving fever management.

Our model suggests that the single most important intervention to increase the overall percentage of all febrile cases managed correctly and all malaria cases in the community treated with ACTs would be to improve attendance within 24 hours at a health facility. Considering only those who attend a primary health facility, increased ACT stock levels was the most critical intervention in potentially improving in the proportion of febrile malaria cases receiving treatment with ACTs. In contrast, the greatest predicted reduction in NMFI cases being overtreated following a single health system intervention was following improved compliance with diagnostic results; although this was anticipated to be associated with a reduction in the proportion of malaria cases receiving ACTs. Multi-pronged intervention strategies were most effective in balancing possible improvements in malaria treatment with the risks of NMFI overtreatment. However substantial improvements in malaria case treatment were not achieved as model outputs without increasing ACT stock levels. Interventions targeted at diagnostic tool availability, use and compliance may improve NMFI management rather than significantly impacting on malaria treatment.

In Tanzania despite reported improved access to diagnostics and compliance with their results as well as expanded ACT stocks, the proportion of malaria cases treated with ACTs is predicted by the model to have reduced following rollout of new WHO guidelines, whilst levels of NMFI overtreatment are predicted to have decreased. This is due to an anticipated large reduction in the numbers of malaria cases that receive ACTs despite being untested or who test falsely negative. Our model did not differentiate between the likelihood in receiving ACTs if untested due to healthcare choice or lack of diagnostic availability. However, this model output highlights the need for improved quality of testing, and also proper communication of the new WHO guidance to HCWs to prevent any malaria under-treatment if diagnostics are unavailable. Of note our analysis did not include patients receiving antimalarials other than ACTs.

Health system interventions for case management of malaria must be guided by whether the priority is improvement in malaria cases receiving ACTs, i.e. reducing the treatment gap, reducing ACT waste through unnecessary treatment of NMFI, i.e. treatment excess, increasing appropriate treatment of all febrile illness or expanding the most cost-effective solution for that particular epidemiological environment. This has implications for the recent emphasis on rollout of RDTs and the WHO guidance, but also highlights the need to focus on stock-management and improving HCW training in diagnostics. These priorities and the most cost-effective way to manage fevers may vary by transmission setting. Lubell et al, used a decision-tree cost modelling approach to suggest that use of diagnostics at moderate and low levels of transmission was more cost-beneficial than presumptive treatment (providing compliance to test results was high), but that this was less clear in high transmission settings [38]. We found a paucity of data on case management indicators in low malaria prevalence settings, but our results mirror intuitive assumptions that the high levels of diagnostic use and compliance with results may have an important role to play here in reducing levels of overtreatment with ACTs in NMFI cases.

A limitation of our decision-tree approach is the assumption that the parameters are independent of each other. It would seem likely that the availability and use of diagnostics are related to each other, and stock levels of ACTs may also influence whether testing occurs, but there is little data to parameterise such an association. We did not include staff training in this analysis at this stage, as there is much uncertainty about the impact of training on HCW performance [28], [30], [42]. In addition we used the same probability of receiving ACTs when untested irrespective of the presence of diagnostics which may not reflect reality and will need further study of HCW behaviour. From a published systematic review [40], we used aggregated data from several countries from across Africa (and outside Africa for a low prevalence scenario), but these are unlikely to be comparable, and fail to provide specific guidance to nuanced health systems setting. Data from Tanzania alone gave a similar pattern of results; however the majority of the aggregated data was also from East Africa. There was substantial variation in data collection methods, sample sizes and the nature of the data collected. Despite these limitations, our results demonstrate the feasibility of such a decision-tree approach to quantify the effects of investing in changing health systems parameters, which could be made site-specific if such data were available.

Further work is required to explore the most cost-effective targets to expand the delivery of antimalarials and reduce ACT waste, given limited malaria control budgets and the potential rise of ACT resistance. In addition, this approach could be extended to delivery through other sectors including community HCWs and the private drug shops. This would be a useful tool with which to reflect on the impact of private sector subsidy schemes such as the Affordable Medicines Facility for malaria (AMFm) [43]. It will also be critical to investigate if improving access to and the performance of health systems may allow reductions in malaria transmission intensity and disease mortality and morbidity. As malaria transmission declines and appropriate treatment for NMFI becomes of increasing importance, it will become necessary to adopt a holistic approach to investing in improving fever management, both malaria and NMFI, taking into consideration the particular characteristics of the health systems, including the contributions of public, private and community delivery.
